# Intestinal permeability and inflammation mediate the association between nutrient density of complementary foods and biochemical measures of micronutrient status in young children: results from the MAL-ED study

**DOI:** 10.1093/ajcn/nqz151

**Published:** 2019-08-06

**Authors:** Benjamin J J McCormick, Laura E Murray-Kolb, Gwenyth O Lee, Kerry J Schulze, A Catharine Ross, Aubrey Bauck, Aldo A M Lima, Bruna L L Maciel, Margaret N Kosek, Jessica C Seidman, Ramya Ambikapathi, Anuradha Bose, Sushil John, Gagandeep Kang, Ali Turab, Estomih Mduma, Pascal Bessong, Sanjaya K Shrestra, Tahmeed Ahmed, Mustafa Mahfuz, Maribel Paredes Olortegui, Zulfiqar Bhutta, Laura E Caulfield, MAL-ED Network Investigators, Angel Mendez Acosta, Rosa Rios de Burga, Cesar Banda Chavez, Julian Torres Flores, Maribel Paredes Olotegui, Silvia Rengifo Pinedo, Mery Siguas Salas, Dixner Rengifo Trigoso, Angel Orbe Vasquez, Imran Ahmed, Didar Alam, Asad Ali, Zulfiqar A Bhutta, Shahida Qureshi, Muneera Rasheed, Sajid Soofi, Ali Turab, Anita K M Zaidi, Ladaporn Bodhidatta, Carl J Mason, Sudhir Babji, Anuradha Bose, Ajila T George, Dinesh Hariraju, M Steffi Jennifer, Sushil John, Shiny Kaki, Gagandeep Kang, Priyadarshani Karunakaran, Beena Koshy, Robin P Lazarus, Jayaprakash Muliyil, Mohan Venkata Raghava, Sophy Raju, Anup Ramachandran, Rakhi Ramadas, Karthikeyan Ramanujam, Anuradha Bose, Reeba Roshan, Srujan L Sharma, Shanmuga Sundaram E, Rahul J Thomas, William K Pan, Ramya Ambikapathi, J Daniel Carreon, Vivek Charu, Viyada Doan, Jhanelle Graham, Christel Hoest, Stacey Knobler, Dennis R Lang, Benjamin J J McCormick, Monica McGrath, Mark A Miller, Archana Mohale, Gaurvika Nayyar, Stephanie Psaki, Zeba Rasmussen, Stephanie A Richard, Jessica C Seidman, Vivian Wang, Rebecca Blank, Michael Gottlieb, Karen H Tountas, Caroline Amour, Eliwaza Bayyo, Estomih R Mduma, Regisiana Mvungi, Rosemary Nshama, John Pascal, Buliga Mujaga Swema, Ladislaus Yarrot, Tahmeed Ahmed, A M Shamsir Ahmed, Rashidul Haque, Iqbal Hossain, Munirul Islam, Mustafa Mahfuz, Dinesh Mondal, Fahmida Tofail, Ram Krishna Chandyo, Prakash Sunder Shrestha, Rita Shrestha, Manjeswori Ulak, Aubrey Bauck, Robert E Black, Laura E Caulfield, William Checkley, Margaret N Kosek, Gwenyth Lee, Kerry Schulze, Pablo Peñataro Yori, Laura E Murray-Kolb, A Catharine Ross, Barbara Schaefer, Suzanne Simons, Laura Pendergast, Cláudia B Abreu, Hilda Costa, Alessandra Di Moura, José Quirino Filho, Alexandre Havt, Álvaro M Leite, Aldo A M Lima, Noélia L Lima, Ila F Lima, Bruna L L Maciel, Pedro H Q S Medeiros, Milena Moraes, Francisco S Mota, Reinaldo B Oriá, Josiane Quetz, Alberto M Soares, Rosa M S Mota, Crystal L Patil, Pascal Bessong, Cloupas Mahopo, Angelina Maphula, Emanuel Nyathi, Amidou Samie, Leah Barrett, Rebecca Dillingham, Jean Gratz, Richard L Guerrant, Eric Houpt, William A Petri, James Platts-Mills, Rebecca Scharf, Binob Shrestha, Sanjaya Kumar Shrestha, Tor Strand, Erling Svensen

**Affiliations:** 1 Fogarty International Center, NIH, Bethesda, MD, USA; 2 Department of Nutritional Sciences, The Pennsylvania State University, University Park, PA, USA; 3 Department of International Health, The Johns Hopkins Bloomberg School of Public Health, Baltimore, MD, USA; 4 Universidade Federal do Ceará, INCT—Instituto de Biomedicina do Semiárido Brasileiro, Fortaleza, Brazil; 5 Department of Nutrition, Federal University of Rio Grande do Norte, Natal, Rio Negro, Brazil; 6 Division of Gastrointestinal Sciences, Christian Medical College, Vellore, India; 7 Centre of Excellence in Women and Child Health, Aga Khan University, Karachi, Pakistan; 8 Haydom Lutheran Hospital, Haydom, Tanzania; 9 University of Venda, Thohoyandou, South Africa; 10 Walter Reed, Armed Forces Research Institute of Medical Sciences (AFRIMS) Research Unit, Nepal (WARUN), Kathmandu, Nepal; 11 Division of Nutrition and Clinical Services, icddr,b, Dhaka, Bangladesh; 12 AB Prisma, Iquitos, Peru; 13 Aga Khan University, Karachi, Pakistan; 14 Armed Forces Research Institute of Medical Sciences, Bangkok, Thailand; 15 Christian Medical College, Vellore, India; 16 Duke University, Durham, NC, USA; 17 Foundation for the NIH, Bethesda, MD, USA; 18 icddr, b, Dhaka, Bangladesh; 19 Institute of Medicine, Tribhuvan University, Kathmandu, Nepal; 20 Johns Hopkins University, Baltimore, MD, USA; 21 The Pennsylvania State University, University Park, PA, USA; 22 Temple University, Philadelphia, PA, USA; 23 Universidade Federal do Ceara, Fortaleza, Brazil; 24 University of Illinois, Chicago, IL, USA; 25 University of Virginia, Charlottesville, VA, USA; 26 Walter Reed/AFRIMS Research Unit, Kathmandu, Nepal; 27 University of Bergen, Norway; 28 Haukeland University Hospital, Bergen, Norway

**Keywords:** environmental enteropathy, intestinal barrier function, inflammation, micronutrient status, diet

## Abstract

**Background:**

Environmental enteric dysfunction (EED) is thought to increase the risk of micronutrient deficiencies, but few studies adjust for dietary intakes and systemic inflammation.

**Objective:**

We tested whether EED is associated with micronutrient deficiency risk independent of diet and systemic inflammation, and whether it mediates the relation between intake and micronutrient status.

**Methods:**

Using data from 1283 children in the MAL-ED (Etiology, Risk Factors, and Interactions of Enteric Infections and Malnutrition and the Consequences for Child Health) birth cohort we evaluated the risk of anemia, low retinol, zinc, and ferritin, and high transferrin receptor (TfR) at 15 mo. We characterized gut inflammation and permeability by myeloperoxidase (MPO), neopterin (NEO), and α-1-antitrypsin (AAT) concentrations from asymptomatic fecal samples averaged from 9 to 15 mo, and averaged the lactulose:mannitol ratio *z*-score (LMZ) at 9 and 15 mo. Nutrient intakes from complementary foods were quantified monthly from 9 to 15 mo and densities were averaged for analyses. α-1-Acid glycoprotein at 15 mo characterized systemic inflammation. Relations between variables were modeled using a Bayesian network.

**Results:**

A greater risk of anemia was associated with LMZ [1.15 (95% CI: 1.01, 1.31)] and MPO [1.16 (1.01, 1.34)]. A greater risk of low ferritin was associated with AAT [1.19 (1.03, 1.37)] and NEO [1.22 (1.04, 1.44)]. A greater risk of low retinol was associated with LMZ [1.24 (1.08, 1.45)]. However, MPO was associated with a lower risk of high transferrin receptor [0.86 (0.74, 0.98)], NEO with a lower risk of low retinol [0.75 (0.62, 0.89)], and AAT with a lower risk of low plasma zinc [0.83 (0.70, 0.99)]. Greater nutrient intake densities (vitamins A and B6, calcium, protein, and zinc) were negatively associated with EED. Inverse associations between nutrient densities and micronutrient deficiency largely disappeared after adjustment for EED, suggesting that EED mediates these associations.

**Conclusions:**

EED is independently associated with an increased risk of low ferritin, low retinol, and anemia. Greater nutrient density from complementary foods may reduce EED, and the control of micronutrient deficiencies may require control of EED.

## Introduction

Micronutrient deficiencies affect millions of young children in low- and middle-income countries and compromise their growth and well-being (1, 2). Major causes of micronutrient deficiencies are inadequate dietary intake and impairments in absorption and modulation of tissue availability of the nutrient to meet metabolic requirements (3).

Repeated enteropathogen infections or subclinical infections can damage the gut and produce a condition called environmental enteric dysfunction (EED) (4, 5), characterized by abnormal morphology (altered crypt depth and villus height, diagnosed via biopsy) (5, 6). For several decades, a noninvasive test of EED, the lactulose:mannitol (LM) test of intestinal absorption, permeability, and damage, has been used to study EED among children. Recently, researchers have also utilized fecal and blood biomarkers of the immune system to characterize additional aspects of EED (7, 8). It is proposed that EED, due to changes in gut architecture and increases in both local gut and subsequent systemic inflammation, may be associated with micronutrient deficiencies, through impaired nutrient absorption, increased requirements, nutrient sequestration, or a combination thereof (9).

Studies that directly evaluate the impact of altered gut function on micronutrient absofrption in children are rare (9). Manary et al. (10) found that increased gut permeability in Malawian children aged 3–5 y (assessed using the LM test) was positively associated with endogenous fecal zinc but negatively with net zinc retention, suggesting altered zinc homeostasis (10). Studies evaluating associations between intestinal permeability and indicators of micronutrient status in children are also limited. In cross-sectional studies, children with a higher LM ratio had lower serum iron and/or were more likely to have anemia (11, 12), and an increased concentration of calprotectin, a fecal biomarker of neutrophil activation, has been associated with lower plasma zinc concentrations (13, 14).

Proteins, vitamin A, and zinc are critical nutrients for maintaining epithelial integrity and promoting immune responses essential for gut health (15), and a low intake of these and other nutrients may exacerbate EED. In previous studies of diet and systemic inflammation, diets providing more vitamin A and zinc (along with vitamins C, D, and E and other antioxidants) have been considered anti-inflammatory (16). In contrast, iron fortification has been reported to increase the LM ratio (i.e., exacerbating EED) (17) and intestinal inflammation (18), and excess dietary iron is hypothesized to be proinflammatory because it may alter gut microbial populations and, in turn, lead to increased morbidity and systemic inflammation (19–21).

The “Etiology, Risk Factors, and Interactions of Enteric Infections and Malnutrition and the Consequences for Child Health” (MAL-ED) 8-site birth cohort study (22) was designed to study the impact of diet, morbidity, and gut function on child growth, cognitive development, and vaccine response (23–25). Here, we use longitudinal measures of diet and gut function to evaluate whether intestinal permeability and inflammation [which are influenced by exposure to enteropathogens (8)] affect the risk of micronutrient deficiency in children aged 15 mo, adjusting for dietary intake and other factors.

## Methods

The MAL-ED study was conducted in 8 populations: Dhaka, Bangladesh (BGD); Fortaleza, Brazil (BRF); Vellore, India (INV); Bhaktapur, Nepal (NEB); Loreto, Peru (PEL); Naushero Feroze, Pakistan (PKN); Venda, South Africa (SAV); and Haydom, Tanzania (TZH) (22). Briefly, infants were enrolled within 17 days of birth, if they were born singleton with a birth weight >1500 g, without serious illnesses, to a mother aged ≥16 y, and to a family planning to stay in the community for ≥6 mo. Children were followed until aged 24 mo. Each site enrolled children with the goal of obtaining information until 24 mo on ∼200 children, accounting for loss to follow-up. Each site obtained ethical approval from their respective institutions, and written consent was obtained from the parents.

Here, we focus on the period from 9 to 15 mo of age, because it is a period of risk of the development of micronutrient deficiencies due to inadequate nutrient intake from complementary foods and because of the temporal alignment of the key exposures of dietary intake, gut permeability, and inflammation leading to the end point of micronutrient status at 15 mo. The methods of data collection for infant feeding, dietary intakes and micronutrient status, disease surveillance, and stool microbiology are described elsewhere (26–28) and brief details are provided below (the timeline of data collection is described in **Supplemental Figure 1**).

Starting at enrollment, and through twice-weekly surveillance visits, caregivers were asked about breastfeeding and nonbreast milk food consumption (26). Mothers also reported whether the child had experienced illness in the preceding days (27). Monthly, the mother/caregiver was interviewed to further characterize breastfeeding and the introduction of complementary foods. When children reached 9 mo of age, their energy, macro-, and micronutrient intake from nonbreast milk foods were estimated monthly using a quantitative 24-h recall (26). Breast milk intake was not quantified.

Caregivers were also queried about provision of nutritional supplements to the child through the healthcare system. For analyses, reported supplements were categorized broadly as containing: *1*) iron; *2*) multivitamins with or without minerals (MVM); *3*) zinc; *4*) vitamin B complex or folate; *5*) vitamin D; *6*) vitamin A; *7*) herbals; *8*) amino acids; and*9*) multiple micronutrients in powders (MNP) (BGD and PEL only).

To characterize aspects of gut function that are indicative of EED, normal, i.e., not diarrheal, stools were collected for analysis monthly during the first year and quarterly in the second year. From these stool samples, 3 biomarkers were analyzed: myeloperoxidase (MPO), neopterin (NEO), and α-1-antitrypsin (AAT) (28). Briefly, MPO is a marker of neutrophil activation, NEO indicates T-helper cell activity, and AAT is a marker for protein loss and intestinal permeability.

To evaluate intestinal barrier function, children were administered the LM test aged 9 and 15 mo; test administration and laboratory procedures are reported elsewhere (28). From the test, urinary recovery of lactulose (%) and mannitol (%) were determined as well as their ratio. We have previously demonstrated the importance of normalizing the values to a reference to remove differences due to age and sex (29); therefore, the results are expressed as *z*-scores referred to as lactulose *z*-score (%Lac-Z), mannitol *z*-score (%Man-Z), and their ratio as LMZ.

Venous blood samples were collected at 15 mo to characterize the status of vitamin A (plasma retinol), zinc (plasma zinc), and iron [plasma ferritin and transferrin receptor (TfR)]. A finger-prick blood sample was also obtained to determine hemoglobin concentration using the HemoCue method (30). Retinol was determined using HPLC (31) and plasma zinc was determined using atomic absorption spectrophotometry (32). Plasma ferritin and TfR were determined using immunoturbidimetry (BGD and PKN; Hitachi 902 analyzer), chemiluminescence (INV; Hitachi 912 analyzer), or by enzyme immunoassay calibrated against WHO-certified values (all other sites; Ramco Laboratories Inc.). Plasma α-1-acid glycoprotein (AGP) as a measure of systemic inflammation was determined by autoanalyzers (as above) or using radial immunodiffusion (Kent Laboratories); it was considered elevated at concentrations >1 g/L. Laboratories participated in global laboratory standardization programs using Standard Reference Materials produced by the National Institute of Standards and Technology, and used quality control materials with certified concentrations of analytes of interest in a human serum or plasma matrix to ensure assay quality and agreement across sites.

To report the prevalence of micronutrient deficiencies, we adjusted the distributions of each biochemical indicator for inflammation per Biomarkers Reflecting Inflammation and Nutritional Determinants of Anemia guidelines (33). Vitamin A deficiency was defined as a retinol concentration <0.70 μmol/L, zinc deficiency as a zinc concentration <9.9 μmol/L, iron deficiency as a plasma ferritin concentration <12 μg/L, and TfR was defined as elevated if the concentration was >8.3 mg/L. Hemoglobin was adjusted for altitude where appropriate (NEB and TZH) (34), and anemia was identified by hemoglobin <11.0 g/dL. The plasma zinc concentrations for the SAV site indicated contamination in initial sample processing and because we planned to examine associations amongst indicators of micronutrient deficiency simultaneously, we therefore excluded the SAV data from these analyses.

### Sample size and statistical analyses

**FIGURE 1:**
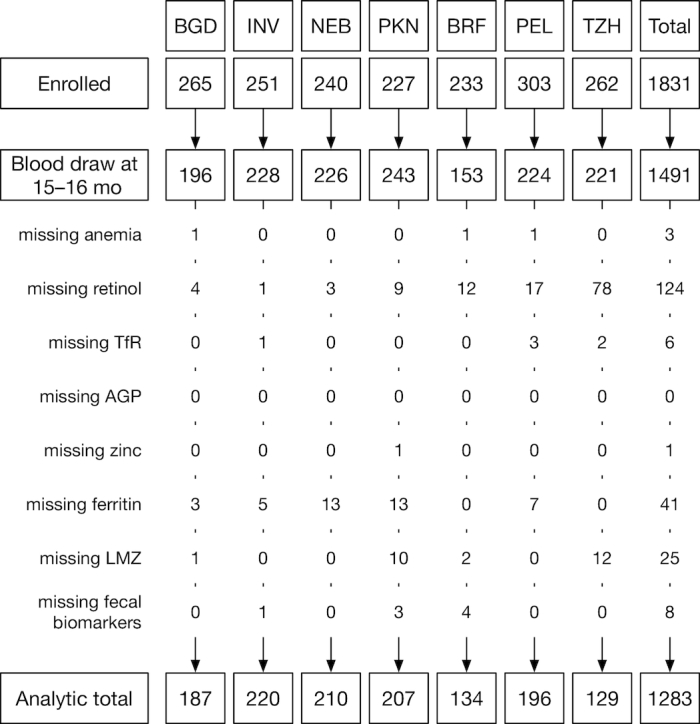
Cohort profile by site. AGP, α-1-acid glycoprotein; BGD, Dhaka, Bangladesh; BRF, Fortaleza, Brazil; INV, Vellore, India; LMZ, lactulose:mannitol ratio *z*-score; NEB, Bhaktapur, Nepal; PEL, Loreto, Peru; PKN, Naushero Feroze, Pakistan; TfR, transferrin receptor; TZH, Haydom, Tanzania.

Of the 1831 children enrolled across the 7 sites, 1574 (86%) remained in the study at 15 mo, and of those, 1491 (95%) had a blood draw at 15 (or 16) mo (**Figure 1**). The completeness of the biochemical determinations for some children was limited by sample volume and condition; the protocol prioritized the assays based on potential associations with the primary outcomes of child growth and development, and plasma retinol had the lowest priority. Of the 175 children with incomplete micronutrient status data, 124 (71%) did not have a plasma retinol determination. From 9 to 15 mo, a maximum of 7 assessments of intake from complementary foods, 5 assessments of gut function, and 2 assessments of barrier function were available. Thirty-one children had missing data for other key variables, resulting in a final analytic sample size of 1283 children (assuming complete-cases analysis, 82% of children in the study at 15 mo, ranging from 55% in TZH to 91% in NEB).

The concentration of the 3 fecal biomarkers were log-transformed and the mean was then calculated over observations from 9 to 15 mo. The mean of the 2 urinary %Lac-Z, %Man-Z, and LMZ measurements at 9 and 15 mo were also calculated with the interpretation of a higher LMZ as indicative of greater gut dysfunction, a higher %Lac-Z of greater permeability, and a higher %Man-Z as greater surface area for absorption.

Mean intake from nonbreast milk foods was calculated from 9 to 15 mo. To describe the dietary pattern, macronutrient intake is presented by the % contribution to energy intake. During analyses, we evaluated macro- and micronutrient densities (intake per 1000 kcal and subsequently transformed using a square-root function), including the intake of protein, vitamins (A, B6, B12, C, and folate), and minerals (calcium, zinc, and iron). Breastfeeding is described by the proportion of days a child was breastfed between 9 and 15 mo.

Univariate associations between nutrient density (square-root transformed and standardized to mean 0, SD 1) and the micronutrient status variables (binary) or the markers of EED and inflammation (on a log scale, then standardized) were calculated using generalized mixed models including site as a random intercept.

**FIGURE 2:**
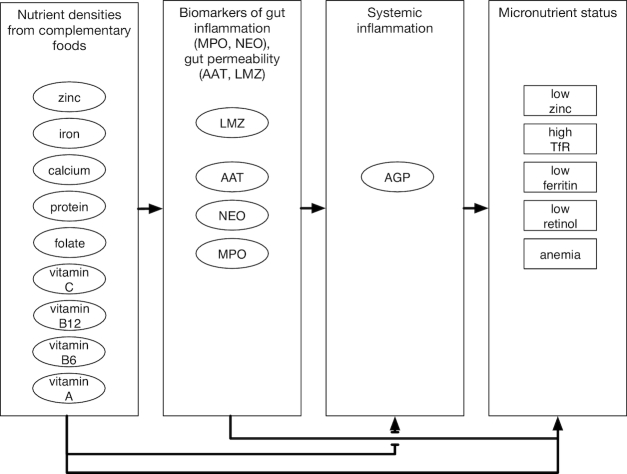
Schematic of the model used to examine hypothetical relations between variables. Nutrient intake densities from complementary foods are related to fecal biomarkers of inflammation (MPO, myeloperoxidase; NEO, neopterin) to markers of permeability (AAT, α-1-antitrypsin; LMZ, lactulose:mannitol ratio *z*-score) and to plasma biomarkers of nutrient status, which are also affected by systemic inflammation (AGP, α-1-acid glycoprotein). The plasma biomarkers (including AGP) were measured at 15 mo, whereas all other variables are the averages from 9 to 15 mo (LMZ was measured at 9 and 15 mo only). TfR, transferrin receptor.

Bayesian network analysis allows the evaluation of statistical associations amongst variables including one or more outcome measures (35, 36). Using the conceptual framework (**Figure 2**), a Bayesian network (35, 37) was developed to document hypothesized relations between diet and micronutrient status, diet and gut inflammation, permeability and systemic inflammation, and their associations with micronutrient status. Because we were interested in evaluating the association of AGP with other variables, we utilized noninflammation-adjusted biochemical values and explicitly included (log-transformed) AGP in the model. The Bayesian network was constructed as conditionally independent generalized linear regressions using JAGS 4.2.0 (http://mcmc-jags.sourceforge.net)(38). Coefficients are interpreted as the mean effect of a unit change in an ancestor (a predictor variable) from which an arc (arrow) originates on a descendent (dependent variable) where the arc ends, as one would with a linear regression. We hypothesized a mediating role of the gut function biomarkers such that relations between nutrient intake and micronutrient status would be determined (at least partially) by degrees of gut inflammation and permeability. Each node (i.e., the micronutrient outcomes and gut function variables) included additional dummy variables to adjust for site. To fit the model, the diet, gut biomarker, LMZ, and AGP variables were standardized. Mean effects (for continuous biomarkers) and OR (for binary micronutrient status biomarkers) per 1 SD difference and 95% credibility interval are reported for selected results.

### Sensitivity analyses

From 9 to 15 mo as well as in the days proximate to the blood draw at 15 mo, fever as reported by the mother was recorded. Three variables (proportion of days with fever 9–15 mo; fever <7 d prior – or fever <7 d following – the blood draw) were included in generalized linear mixed models as additional markers of systemic inflammation in order to evaluate their influence on the risk of the micronutrient status outcomes and on the other coefficients in the model.

The provision of nutritional supplements to children varied widely across the sites; for example, between 9 and 15 mo, 87% of children in PEL received iron supplements, but no supplements were reported for TZH or BRF. We conducted sensitivity analyses to evaluate the impact of including variables for MVM and MNP, and iron and zinc supplements in the Bayesian network. Provision of iron supplements was associated with a lower risk of anemia at 15 mo in PEL, but it did not affect the observed relations amongst diet, gut function, and micronutrient status. Therefore, we did not adjust for them in the Bayesian network presented here.

## Results

Characteristics of the subset of the MAL-ED population included in these analyses are shown for biomarkers of gut function and micronutrient status in **Table 1**. Each of the fecal biomarkers and LMZ were elevated when compared with available reference data (29, 39). Children were reported to spend 0.5–10.8% days with fever, at 15 mo, 40–69% of children had an elevated AGP concentration, and 5–35% of children had fever near the time of the blood draw (**Supplemental Table 1**). The prevalence of anemia ranged from 40% in BRF to 88% in PKN. Prevalence of other micronutrient deficiencies differed similarly across sites: low plasma retinol ranged from 6% in BRF to 61% in TZH; and low plasma zinc ranged from 2% in BRF to 73% in INV. The prevalence of high TfR ranged from 11% in TZH to 72% in BRF, but the prevalence of low plasma ferritin was more similar across sites, affecting 51% of children in most sites, although as much as 82% in PKN. Ferritin concentration was negatively correlated with TfR (mean Spearman's ρ: −0.32 [95% CI: −0.37, −0.26]) and positively correlated with hemoglobin concentration [0.40 (0.35, 0.44)], whereas TfR was negatively correlated with hemoglobin concentration [−0.24 (−0.28, −0.19)]. The 3 fecal biomarkers were correlated with each other (ρ ∼0.3; **Supplemental Figure 2**) but less so with LMZ and AGP (ρ ∼0.1–0.2).

**TABLE 1:** Indicators of gut function, micronutrient status, and inflammation by MAL-ED study site^1^

	BGD	INV	NEB	PKN	BRF	PEL	TZH
Children in analysis, *n*	187	220	209	207	133	196	129
Urinary biomarkers							
%Lac-Z^2^	1.5 ± 0.74	2.3 ± 0.77	0.81 ± 0.72	1.1 ± 1.1	0.047 ± 0.74	1.5 ± 0.81	0.092 ± 1.3
%Man-Z^2^	0.87 ± 0.68	1.2 ± 0.76	0.66 ± 0.87	0.35 ± 1	0.016 ± 0.78	0.23 ± 0.69	−0.19 ± 0.99
LMZ^2^	0.29 ± 0.67	0.84 ± 0.67	0.062 ± 0.8	0.68 ± 0.84	0.039 ± 0.74	1 ± 0.54	0.39 ± 1.3
Fecal biomarkers							
MPO ln(ng/mL)^3^	8.5 ± 0.8	8.8 ± 0.65	8.5 ± 0.74	8.1 ± 0.88	8 ± 1.2	8.8 ± 0.88	8.2 ± 0.76
NEO ln(nmol/L)^3^	6.8 ± 0.82	7.5 ± 0.67	7.6 ± 0.44	6.3 ± 0.58	7.4 ± 0.71	7.9 ± 0.53	6.5 ± 0.95
AAT ln(μg/g)^3^	−1 ± 0.61	−1.1 ± 0.65	−0.93 ± 0.57	−2 ± 0.78	−1.4 ± 0.75	−0.84 ± 0.59	−1.4 ± 0.86
Biomarkers of micronutrient status							
Hemoglobin, g/dL^4^	11 ± 1.5	11 ± 1.3	11 ± 1.2	9.3 ± 1.4	11 ± 2.2	11 ± 1.2	11 ± 1.2
Retinol, μmol/L^5^	27.2 ± (9)	31.4 ± 9.5	30.4 ± 7.5	21.4 ± 8.3	35.1 ± 9.7	24.9 ± 6.9	18.9 ± 6.4
TfR, mg/L^5^	7.2 ± 3.7	4.9 ± 3.6	9.5 ± 4.3	7 ± 5.1	9.9 ± 2.5	7.4 ± 2.6	5.1 ± 3
Ferritin, μg/L^5^	12.7 ± 9.4	10.5 ± 13.3	10.2 ± 36	9.2 ± 14.2	15.1 ± 9.2	17.1 ± 19.5	10.8 ± 9.3
Zinc, μmol/L^5^	11.5 ± 1.7	9.2 ± 1.3	12.3 ± 5.2	8.5 ± 2.6	14.9 ± 4	17 ± 6.6	11.6 ± 2.5
Inflammation							
AGP g/L	0.96 ± 0.32	0.99 ± 0.36	1.20 ± 0.41	0.98 ± 0.41	1.10 ± 0.37	1.20 ± 0.45	1.20 ± 0.40
Inflamed, %^6^	42.0	40.1	66.2	43.3	48.5	66.5	68.8
Fever, %	6.6 (4.4, 9.4)	5.6 (3.8, 8.5)	4.7 (2.3, 7.5)	10.8 (7, 16)	0.5 (0, 0.9)	4.2 (2.3, 6.1)	1.9 (0.5, 3.3)

1 Values are mean ± SD except for the % follow-up of fever (9–15 mo), which is the median (IQR). AAT, α-1-antitrypsin; AGP, α-1 acid glycoprotein; BGD, Dhaka, Bangladesh; BRF, Fortaleza, Brazil; INV, Vellore, India; %Lac-Z, lactulose recovery *z*-score; LMZ, lactulose:mannitol ratio *z*-score; %Man-Z, mannitol recovery *z*-score; MAL-ED, Etiology, Risk Factors, and Interactions of Enteric Infections and Malnutrition and the Consequences for Child Health; MPO, myeloperoxidase; NEB, Bhaktapur, Nepal; NEO, neopterin; PEL, Loreto, Peru; PKN, Naushero Feroze, Pakistan; TfR, transferrin receptor; TZH, Haydom, Tanzania.

2 Mean *z*-scores controlling for age and sex from 9 and 15 mo old, centered on the BRF data.

3 Mean natural log-transformed concentrations from 9 to 15 mo.

4 Hemoglobin adjusted for altitude where applicable (NEB and TZH).

5 Values at 15 mo adjusted for inflammation [AGP, see (33)].

6 Percentage of children with AGP ≥1.0 g/L

The median energy intake from complementary foods (9–15 mo inclusive) differed among the 7 sites, ranging from 240 kcal/d in BGD to 880 kcal/d in BRF (**Table 2**). These differences were driven by the site-to-site variations in infant feeding practices, the introduction of nonbreast milk foods prior to 6 mo, and differences in continued breastfeeding through to 15 mo. Between 53% and 70% of the energy consumed from complementary foods was from carbohydrates, ∼11–17% from protein, and the remainder from fat. The nutrient densities were correlated with one another to varying degrees (ρ ∼0.1–0.7, Supplemental Figure 2).

**TABLE 2:** Usual intakes of energy and macro- and micronutrients from complementary foods at 9–15 mo by MAL-ED study site^1^

	BGD	INV	NEB	PKN	BRF	PEL	TZH
Energy, kcal	240 ±120	580 ± 250	300 ± 130	460 ± 230	880 ± 270	510 ± 180	850 ± 220
Carbohydrate, %E	64 ± 6.8	61 ± 6.5	58 ± 7.7	53 ± 8.7	53 ± 3.9	70 ± 5.7	65 ± 6.8
Protein, %E	12 ± 1.9	11 ± 1.6	11 ± 2.4	11 ± 2	17 ± 2.3	11 ± 2.2	12 ± 1.8
Fat, %E	20 ± 5.4	24 ± 6.3	27 ± 7.1	33 ± 8.8	28 ± 3.2	19 ± 4.5	19 ± 5.9
Nutrient densities (per 1000 kcal)							
Iron, mg	4.1 (3.2, 4.8)	3.4 (3, 3.7)	3.6 (2.6, 4.1)	3.3 ±(2.3, 4.1)	14 (11, 17)	5.9 (3.2, 6.2)	7.7 (6.8, 8.5)
Zinc, mg	3.5 (3.1, 3.9)	4 (3.6, 4.4)	3.5 (3, 4)	3.3 (2.9, 3.6)	9.2 (7.9, 11)	3.1 (2.6, 3.4)	5.5 (5.2, 5.7)
Calcium, mg	320 (150, 450)	440 (220, 640)	330 (180, 450)	550 (360, 710)	1100 (1000, 1200)	360 (170, 470)	490 (270, 690)
Vitamin A, μg	190 (94, 250)	230 (130, 330)	210 (130, 280)	250 (160, 320)	1100 (940, 1200)	430 (200, 500)	170 (110, 210)
Vitamin B6, μg	0.8 (0.7, 1.0)	0.4 (0.3, 0.5)	0.6 (0.5, 0.7)	0.7 (0.5, 0.9)	1.2 (0.8, 1.4)	0.5 (0.4, 0.6)	0.1 (0.1, 0.2)
Folate, μg	110 (83, 130)	120 (100, 130)	120 (85, 140)	95 (73, 110)	180 (150, 190)	93 (67, 100)	69 (58, 78)
Vitamin B12, μg	1.5 (0.8, 2)	0.7 (0.4, 1.1)	1.3 (0.7, 1.8)	1.5 (0.8, 2)	5.1 (4.6, 5.3)	1.7 (0.9, 2.1)	1.7 (0.9, 2.5)
Vitamin C, mg	36 (15, 45)	13 (10, 14)	19 (9.2, 23)	19 (10, 23)	120 (88, 130)	110 (27, 120)	5.4 (2.4, 6.9)
Breastfed, proportion of days	1 (1, 1)	1 (0.7, 1)	1 (1, 1)	1 (0.9, 1)	1 (0.5, 1)	1 (1, 1)	1 (1, 1)

1 Intakes are based on the means of ≤7 monthly measures of intake per child. Energy and macronutrient intakes expressed as mean ± SD, and macronutrients are expressed as % of energy (%E). Micronutrient densities per 1000 kcal are shown as median (25th, 75th percentile). BGD, Dhaka, Bangladesh; BRF, Fortaleza, Brazil; INV, Vellore, India; MAL-ED, Etiology, Risk Factors, and Interactions of Enteric Infections and Malnutrition and the Consequences for Child Health; NEB, Bhaktapur, Nepal; PEL, Loreto, Peru; PKN, Naushero Feroze, Pakistan; TZH, Haydom, Tanzania.

Adjusting for inflammation and site, nutrient densities from complementary foods as well as biomarkers of EED were associated with the risk of micronutrient deficiency (**Table 3**). Greater vitamin A nutrient density was associated with a lower risk of low plasma retinol, and greater iron nutrient density was associated with a lower risk of anemia, low ferritin, and low retinol, but not with a risk of high TfR. No statistically significant associations between diet and zinc status were detected. The risk of anemia and low retinol were associated with greater LMZ, and the risk of low ferritin was associated with AAT. Inverse associations were also observed: NEO with a lower risk of low retinol, MPO with a lower risk of high TfR, and AAT with a lower risk of low plasma zinc.

**TABLE 3:** ORs (95% CI) for micronutrient deficiency associated with a 1-SD change in nutrient density in complementary foods or in biomarkers of EED^1^

Variable	Anemia	Low plasma ferritin	High transferrin receptor	Low plasma retinol	Low plasma zinc
Usual nutrient density, SD [sqrt(intake/1000 kcal)]					
Iron, mg	0.78 (0.64, 0.94)*	0.66 (0.54, 0.79)*	0.88 (0.73, 1.07)	0.71 (0.57, 0.88)*	0.76 (0.55, 1.06)
Zinc, mg	0.89 (0.71, 1.1)	0.80 (0.64, 1.01)	1.05 (0.84, 1.32)	0.59 (0.45, 0.78)*	1.26 (0.88, 1.80)
Calcium, mg	1.04 (0.9, 1.21)	1.02 (0.88, 1.18)	1.19 (1.02, 1.39)*	0.72 (0.61, 0.86)*	1.16 (0.97, 1.39)
Vitamin A, μg	1.22 (1.02, 1.46)*	0.94 (0.78, 1.14)	1.14 (0.95, 1.38)	0.81 (0.67, 1.00)*	1.14 (0.88, 1.47)
Vitamin B6, μg	0.85 (0.71, 1.02)	0.86 (0.71, 1.03)	1.00 (0.82, 1.21)	0.99 (0.80, 1.21)	1.05 (0.82, 1.34)
Folate, μg	1.02 (0.88, 1.17)	0.89 (0.76, 1.02)	0.9 (0.77, 1.04)	0.89 (0.76, 1.05)	0.87 (0.71, 1.07)
Vitamin B12, μg	1.09 (0.93, 1.29)	1.02 (0.86, 1.2)	1.12 (0.94, 1.32)	0.83 (0.69, 0.99)*	1.14 (0.93, 1.41)
Vitamin C, mg	1.05 (0.89, 1.23)	0.84 (0.7, 1.01)	1.04 (0.89, 1.23)	1.06 (0.89, 1.26)	1.01 (0.75, 1.35)
Protein, g	1.02 (0.88, 1.19)	1.09 (0.93, 1.28)	1.04 (0.89, 1.22)	0.85 (0.72, 1.01)	1.18 (0.96, 1.44)
Biomarker of EED					
MPO, SD[log(ng/mL)]	1.10 (0.97, 1.24)	0.97 (0.85, 1.09)	0.85 (0.75, 0.96)*	1.12 (0.97, 1.29)	0.94 (0.80, 1.11)
NEO, SD[log(nmol/L)]	1.00 (0.86, 1.17)	1.19 (1.02, 1.39)*	1.13 (0.96, 1.33)	0.77 (0.66, 0.92)*	1.02 (0.85, 1.23)
AAT, SD[log(μg/g)]	1.00 (0.87, 1.15)	1.13 (0.99, 1.29)*	0.94 (0.82, 1.08)	1.04 (0.90, 1.20)	0.81 (0.69, 0.96)*
LMZ, SD	1.19 (1.03, 1.36)*	0.93 (0.82, 1.07)	0.89 (0.77, 1.02)	1.34 (1.15, 1.57)*	0.98 (0.84, 1.15)

1 Presented are the log odds, adjusted for site and α-1-acid glycoprotein. Nutrient densities were transformed using square root; MPO, NEO, and AAT were transformed using a natural logarithm. * indicates associations that do not include 1.0 in the 95% CI. AAT, α-1-antitrypsin; EED, environmental enteric dysfunction; LMZ, lactulose:mannitol ratio *z*-score; MAL-ED, Etiology, Risk Factors, and Interactions of Enteric Infections and Malnutrition and the Consequences for Child Health; MPO, myeloperoxidase; NEO, neopterin.

In univariate analyses (adjusted for site), the nutrient densities from complementary foods were negatively associated with biomarkers of gut permeability and inflammation, and with systemic inflammation (**Table 4**). Only the zinc density of the diet was negatively associated with LMZ; this association was due to a negative association between zinc density and permeability [%Lac-Z: −0.15 (−0.25, −0.05)]. Based on the univariate results, 2 variables, folate and vitamin C, were dropped from the multivariable model: neither folate nor vitamin C had associations with the micronutrient status or gut function variables, and furthermore, folate was colinear with vitamins B6 and B12 that were retained in the model.

**TABLE 4:** Mean effect of a 1-SD change in nutrient densities from complementary foods on the concentration of biomarkers of environmental enteric dysfunction and systemic inflammation (each of which was also scaled to mean 0, SD 1)^1^

Usual nutrient density, SD [sqrt(intake/1000 kcal)]	MPO	NEO	AAT	LMZ	AGP
Iron, mg	−0.01 (−0.09, 0.07)	−0.04 (−0.11, 0.03)	0.04 (−0.04, 0.12)	−0.05 (−0.13, 0.03)	−0.03 (−0.12, 0.05)
Zinc, mg	−0.09 (−0.19, 0.01)	−0.05 (−0.13, 0.04)	−0.15 (−0.24, −0.05)*	−0.14 (−0.23, −0.04)*	−0.13 (−0.23, −0.04)*
Calcium, mg	−0.15 (−0.21, −0.08)*	−0.08 (−0.13, −0.03)*	−0.19 (−0.25, −0.13)*	−0.04 (−0.11, 0.02)	−0.05 (−0.11, 0.02)
Vitamin A, μg	−0.12 (−0.2, −0.04)*	−0.14 (−0.2, −0.07)*	−0.21 (−0.29, −0.14)*	−0.05 (−0.13, 0.02)	−0.1 (−0.18, −0.02)*
Vitamin B6, μg	−0.03 (−0.11, 0.05)	0.01 (−0.06, 0.08)	−0.04 (−0.11, 0.04)	−0.02 (−0.1, 0.06)	−0.02 (−0.1, 0.06)
Folate, μg	−0.02 (−0.08, 0.04)	−0.02 (−0.07, 0.04)	−0.02 (−0.08, 0.04)	−0.01 (−0.07, 0.06)	−0.08 (−0.15, −0.02)*
Vitamin B12, μg	−0.05 (−0.12, 0.03)	−0.07 (−0.13, −0.01)*	−0.19 (−0.26, −0.13)*	−0.05 (−0.12, 0.02)	−0.08 (−0.15, −0.01)*
Vitamin C, mg	0 (−0.07, 0.08)	0.05 (−0.01, 0.11)	0.01 (−0.06, 0.08)	−0.03 (−0.11, 0.04)	−0.06 (−0.13, 0.02)
Protein, g	−0.12 (−0.19, −0.06)*	−0.01 (−0.07, 0.04)	−0.14 (−0.2, −0.07)*	−0.02 (−0.09, 0.04)	−0.08 (−0.15, −0.01)*

1 Nutrient densities were transformed using a square-root function, MPO, NEO, and AAT were transformed using a natural logarithm function and then each, along with AGP, was scaled to mean 0, SD 1. * indicates associations that do not include 0 in the 95% CI. AAT, α-1-antitrypsin; AGP, α-1-acid glycoprotein; LMZ, lactulose:mannitol ratio *z*-score; MPO, myeloperoxidase; NEO, neopterin.

**FIGURE 3:**
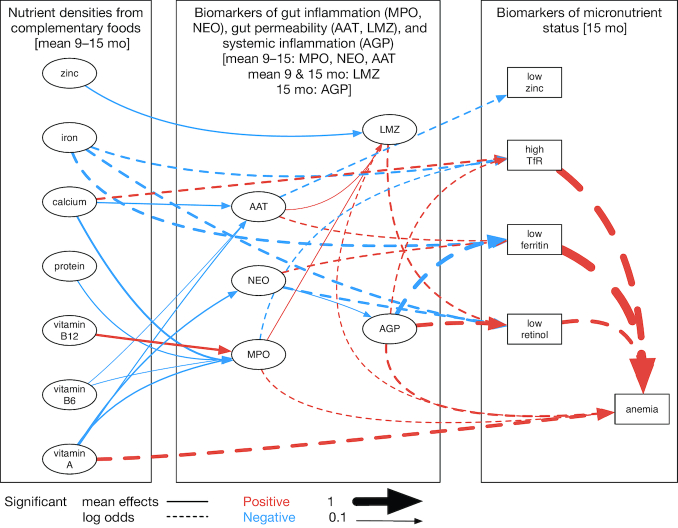
Results of the multivariate Bayesian network. Arcs (arrows) are shown for parameters that did not include zero in the 95th percentile credibility interval. Positive associations are shown in red and negative in blue. The thickness of the arc is proportional to the magnitude of the association, with solid lines indicating linear associations and dashed lines indicating log odds. AAT, α-1-antitrypsin; AGP, α-1 acid glycoprotein; LMZ, urinary lactulose:mannitol ratio *z*-score; MPO, myeloperoxidase; NEO, neopterin; TfR, transferrin receptor.

The results of the Bayesian network are sho wn in **Figure 3** (with all associations shown in **Supplemental Figure 3** and numerical results in **Supplemental Table 2**) with the colors indicating the direction of the association (red, positive; blue, negative) for the relations of diet with gut function biomarkers and AGP, or the log odds for the associations with micronutrient deficiency. Low ferritin, low retinol, and high TfR were each strongly associated with a higher risk of anemia: low ferritin [2.79 (2.09, 3.68)]; low retinol [1.58 (1.16, 2.15)]; high TfR [2.26 (1.68, 3.09)]. However, low zinc concentration was not [0.93 (0.64, 1.29)]. All of the indicators except zinc were also affected by systemic inflammation; higher AGP was associated with a greater risk of low plasma retinol [1.58 (1.38, 1.83)], elevated TfR [1.18 (1.03, 1.33)], and anemia [1.29 (1.12, 1.49)], and with a lower risk of low ferritin [0.62 (0.54, 0.70)].

Biomarkers of gut function were associated with the risk of micronutrient deficiency. A greater risk of anemia was associated with greater intestinal permeability [LMZ: 1.15 (1.01, 1.31)] and inflammation [higher MPO: 1.16 (1.01, 1.34)]. A greater risk of low ferritin was associated with both AAT [1.19 (1.03, 1.37)] and NEO [1.22 (1.04, 1.44)]. Only MPO was associated with a risk of high TfR, and it was protective [0.86 (0.74, 0.98)]. A greater risk of low retinol was also associated with permeability [LMZ: 1.24 (1.08, 1.45)], but inversely with NEO [0.75 (0.62, 0.89)]. Finally, AAT was associated with a lower risk of low plasma zinc [0.83 (0.70, 0.99)]. In general, the pattern of associations between EED and micronutrient deficiencies observed in univariate analyses were also observed in the Bayesian network.

When evaluated within the Bayesian network (Figure 3), all of the dietary associations with AGP were no longer significant. Vitamin A nutrient density remained negatively associated with each of the fecal biomarkers. Calcium and vitamin B6 nutrient densities were each negatively associated with MPO and AAT, and protein density was inversely associated with MPO. Zinc nutrient density remained the only significant (inverse) association with LMZ. Of note, vitamin B12 density emerged in the Bayesian network as positively associated with MPO.

Including associations with markers of gut function and AGP (Figure 3), the association between vitamin A nutrient density and plasma retinol was no longer significant [1.08 (0.80, 1.45)]. However, greater iron density remained associated with a lower risk of low ferritin [0.73 (0.57, 0.92)], and with a lower risk of high TfR [0.79 (0.63, 0.99)] and low retinol [0.74 (0.55, 0.99)]. However, the association with anemia was no longer significant [0.86 (0.66, 1.12)]. The calcium nutrient density of complementary foods was positively associated with elevated TfR, in univariate analyses and in the Bayesian network [1.28 (1.01, 1.61)]. Similarly, vitamin A nutrient density was positively associated with anemia in both sets of analyses [Bayesian network OR: 1.52 (1.13, 2.02)].

Of the 3 additional characterizations of systemic inflammation, only maternally reported fever in the 7 d prior to blood draw was associated with a reduced risk of low ferritin (**Supplemental Figure 4**). Inclusion of these variables did not affect other coefficients in the model (Figure 3) except for a slight reduction in the magnitude of AGP.

## Discussion

The children participating in the MAL-ED study were continuously exposed to multiple enteropathogens (23, 40, 41). The elevated biomarkers of gut function suggest that their gastrointestinal systems experienced varying degrees of inflammation, cellular immune activation, heightened permeability and damage as evidenced by protein loss (39, 42). Although variable across sites, many children had anemia and micronutrient deficiencies. Our results demonstrate that biomarkers of gut function characterizing EED differentiate the risk of micronutrient deficiency independent of dietary intake and systemic inflammation.

The intake of micronutrients, shown here as nutrient densities in complementary foods between 9 and 15 mo of age, differed by site, were inadequate for iron in all sites, and except for BRF, were largely inadequate for vitamin A and zinc (when compared with the estimated average requirements for children). With the exception of vitamin B12, greater nutrient densities from complementary foods were associated with lower intestinal inflammation and permeability, vitamin A density was inversely associated with MPO, NEO, and AAT, and zinc density was inversely associated with LMZ. Greater nutrient densities of iron differentiated the risk of anemia, low ferritin, and low retinol, and greater vitamin A density differentiated the risk of low retinol. However, associations between nutrient density and micronutrient deficiency largely disappeared when biomarkers of EED were considered, suggesting that EED mediated the associations between intake and micronutrient status. Importantly, however, iron density from complementary foods was not associated with any of the gut function markers, and in the Bayesian network, greater iron density remained associated with a lower risk of low ferritin, high TfR, and low retinol, all of which affect the risk of anemia in these children. Unexpectedly, higher vitamin A nutrient density was positively associated with the risk of anemia.

The results related to iron status and anemia are noteworthy. AAT is a large protein, mostly of hepatic origin, which may be present in the gut in minor quantities from ingested breast milk or local synthesis by Paneth cells (43, 44). However, it is a positive acute-phase reactant in systemic circulation and higher mean fecal AAT concentrations indicate loss of AAT from systemic circulation through diffusion, greater permeability, or mucosal damage (45, 46). This raises the question as to whether other plasma proteins may be lost as well. Here, we show that higher fecal AAT is associated with a greater risk of low plasma ferritin, and is also positively associated with LMZ, which is associated with a greater risk of anemia and low retinol. A higher average MPO concentration was also associated with a greater risk of anemia, and this is of interest because MPO is an iron-containing protein released from neutrophils in response to mucosal injury (47), and directly indicates loss of iron from the body. Elevated TfR is an indicator of iron-deficient erythropoiesis; here, we show that TfR is elevated with systemic inflammation, in agreement with other reports (48, 49). That higher average fecal MPO concentration was protective against high TfR may result from cytokine stimulation which activates NEO release and also increases hepcidin production, potentially reducing TfR (47).

Not all of the associations indicated that gut dysfunction was associated with increased risk of micronutrient deficiency. Greater gut permeability (indicated by greater LMZ and greater %Lac-Z) was associated with a lower risk of zinc deficiency, which we postulate reflects greater passive absorption of zinc across the mucosa. Unexpectedly, a higher NEO concentration was associated with a lower risk of low retinol, and our finding is inconsistent with the report by Wieringa et al. (50) of significantly higher circulating NEO concentrations among vitamin A–deficient infants. Further studies of the mechanisms that regulate the production and turnover of both NEO and retinol are needed to elucidate these (or our) results.

This study provides novel findings linking the nutrient density of complementary foods with markers of gut function and inflammation and systemic inflammation. Negative associations between these biomarkers and nutrient densities involving vitamins A and B6, calcium, zinc, iron, and protein were detected during univariate analyses, with a few of these remaining significant in the overall model, after adjustment for multiple relations across dietary and gut function variables, systemic inflammation, and micronutrient status. That protein, zinc, and vitamin A intakes are key nutrients for the maintenance of gut health has been established through preclinical studies (15). Our findings from 7 sites across 3 continents suggest that greater nutrient density from complementary foods may reduce the gut permeability and inflammation of young children living in poverty, who frequently experience bouts of illness and are continuously exposed to enteropathogens.

As noted earlier, other studies have identified associations between selected markers of EED and micronutrient status (11–14); however, none have taken our comprehensive approach of evaluating multiple aspects of diet, gut function, systemic inflammation, and indicators of micronutrient deficiency. We are able to do this because of the in-depth follow-up of these children with multiple assessments of diet, gut function, and micronutrient status. There are limitations, however; first, our model required observations across each of the many variables and this necessitated dropping from analyses some children who were enrolled into the study who did not provide a 15-mo blood sample, or the sample did not allow for all assays. Imputation of data would have been challenging due to site bias in missing values and would have resulted in dubious modeled results. It was unfortunate that we had to remove the SAV data entirely due to previously identified quality control issues related to the plasma zinc samples, and both BRF and TZH were underrepresented in the analytical data set compared with other sites. Second, despite having multiple indicators of gut function, we assessed systemic inflammation using only AGP and only at 1 time point; our additional analyses with reported fever did not yield new insights, but other direct systemic markers of immunological response would perhaps identify linkages with gut function markers not observed here. Third, although most of these children were breastfed, we have no data on breast milk consumption or the nutrient composition of breast milk; thus, our results do not refer to the nutrient density of the total diet, but rather to that from nonbreast milk foods. Finally, it should be noted that we did not evaluate absorption directly, and cannot distinguish whether intestinal inflammation and permeability disrupt nutrient absorption, increase nutrient requirements, and/or result in dysregulation of nutrient metabolism such as occurs with systemic inflammation. Studies of children in which enteropathogen exposure and markers of gut function are characterized, and nutrient absorption is assessed, e.g., using stable isotopes, are needed to provide further clarity (9), as are studies which also assess regulators of micronutrient absorption and metabolism.

In summary, this study provides novel findings on the relation among gut permeability and inflammatory markers of EED and the risk of micronutrient deficiencies in young children across multiple low-resource settings. Greater nutrient densities from complementary foods may reduce intestinal inflammation and permeability, and reduce the risk of micronutrient deficiencies. Apart from dietary intake and systemic inflammation, gut permeability and inflammation affect the risk of micronutrient deficiencies and anemia. These results underscore the need to reduce ubiquitous enteropathogen exposure and EED, along with improving the nutrient density of complementary foods, as a coordinated strategy to reduce micronutrient deficiencies in young children.

## Supplementary Material

nqz151_Supplemental_File

## Notes

The Etiology, Risk Factors and Interactions of Enteric Infections and Malnutrition and the Consequences for Child Health and Development Project (MAL-ED) was carried out as a collaborative project supported by the Bill & Melinda Gates Foundation, the Foundation for the NIH, and the NIH/Fogarty International Center. The sponsor had no role in the analysis and interpretation of the findings reported here.

Supplemental Figures 1–4 and Supplemental Tables 1 and 2 are available from the “Supplementary data” link in the online posting of the article and from the same link in the online table of contents at https://academic.oup.com/ajcn/.

Present address for GOL: the University of Michigan, Ann Arbor, MI, USA.

Present address for AT: the Interactive Research and Development, Karachi, Pakistan.

Present address for RA: Purdue University, West Lafayette, IN, USA.

Present address for A Bauck: Helen Keller International, Dakar, Senegal.

Present address for JCS: the Sabin Vaccine Institute, Washington, DC, USA.

Abbreviations used: AAT, α-1-antitrypsin; AGP, α-1-acid glycoprotein; BGD, Dhaka, Bangladesh; BRF, Fortaleza, Brazil; CI, credibility interval; EED, environmental enteric dysfunction; INV, Vellore, India; LM, lactulose:mannitol; LMZ, lactulose:mannitol ratio *z*-score; MAL-ED, Etiology, Risk Factors, and Interactions of Enteric Infections and Malnutrition and the Consequences for Child Health; MNP, multiple micronutrients in powder; MPO, myeloperoxidase; MVM, multivitamins with or without minerals; NEB, Bhaktapur, Nepal; NEO, neopterin; PEL, Loreto, Peru; PKN, Naushero Feroze, Pakistan; SAV, Venda, South Africa; TfR, transferrin receptor; TZH, Haydom, Tanzania; %Lac-Z, lactulose *z*-score; %Man-Z, mannitol *z*-score.
