# Supplementary material for: Intestinal permeability and inflammation mediate the association between nutrient density of complementary foods and biochemical measures of micronutrient status in young children: results from the MAL-ED study
Source: Am J Clin Nutr. 2019 Aug 6;110(4):1015–25. doi: 10.1093/ajcn/nqz151 (PMC6766446; doi:10.1093/ajcn/nqz151)
Supplement: nqz151_Supplemental_File [file nqz151_supplemental_file.docx]

**Intestinal permeability and inflammation mediate the association between nutrient density of complementary foods and biochemical measures of micronutrient status in young children: results from the MAL-ED study**

**Authors:** Benjamin JJ McCormick, Laura E Murray-Kolb, Gwenyth O Lee, Kerry J Schulze, A Catharine Ross, Aubrey Bauck, Aldo AM Lima, Bruna LL Maciel, Margaret N Kosek, Jessica C Seidman, Ramya Ambikapathi, Anuradha Bose, Sushil John, Gagandeep Kang, Ali Turab, Estomih Mduma, Pascal Bessong, Sanjaya K Shrestra, Tahmeed Ahmed, Mustafa Mahfuz, Maribel Paredes Olortegui, Zulfiqar Bhutta, Laura E Caulfield and the MAL-ED Network Investigators.

**Supplemental Figure 1**: Timeline of sampling protocol. Dots represent samples taken at monthly intervals and the dashed line depicts on-going twice weekly home visits for disease surveillance in which caregivers were queried about medicines or treatments, which included micronutrient (MN) supplements. Abbreviations used: MPO, myeloperoxidase; NEO, neopterin; AAT, α-1 antitrypsin; LM, lactulose:mannitol test; AGP, α-1 acid glycoprotein; Hb, hemoglobin, TfR, transferrin receptor

**
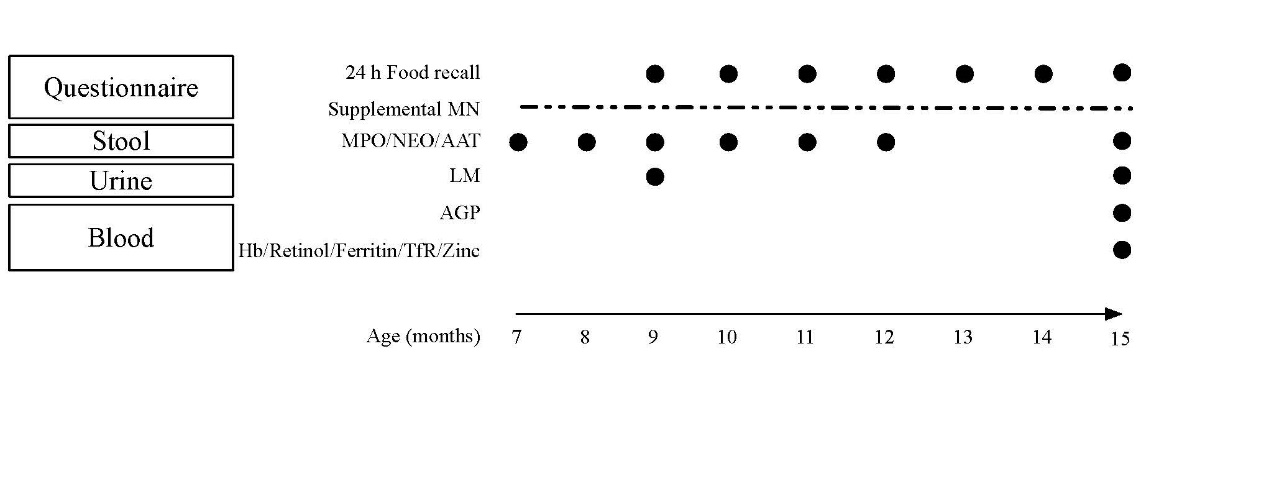
**

**Supplemental Table 1:** Prevalence* of micronutrient deficiencies in the MAL-ED cohort at 15 months old and prevalence of maternally reported fever near the time of the blood draw. Site abbreviations: Dhaka, Bangladesh (BGD); Fortaleza, Brazil (BRF); Vellore, India (INV); Bhaktapur, Nepal (NEB); Loreto, Peru (PEL); Naushero Feroze, Pakistan (PKN); and Haydom, Tanzania (TZH)

| Site | Micronutrient Status  n/N (%) | | | | |  | Maternally reported fever  n/N (%) | |
| --- | --- | --- | --- | --- | --- | --- | --- | --- |
|  | Anemia | Low  Retinol | High  TfR | Low  Ferritin | Low  Zinc |  | <7d before | <7d after |
| BGD | 75/187 (40.1) | 34/174 (19.5) | 54/174 (31) | 94/174 (54) | 30/174 (17.2) |  | 45/187 (24.1) | 32/187 (17.1) |
| INV | 124/220 (56.4) | 22/215 (10.2) | 26/215 (12.1) | 150/215 (69.8) | 156/215 (72.6) |  | 50/220 (22.7) | 52/220 (23.6) |
| NEB | 126/210 (60) | 9/205 (4.4) | 115/205 (56.1) | 165/205 (80.5) | 26/205 (12.7) |  | 35/210 (16.7) | 31/220 (14.8) |
| PKN | 182/207 (87.9) | 98/201 (48.8) | 58/201 (28.9) | 165/201 (82.1) | 147/201 (73.1) |  | 74/207 (35.7) | 73/207 (35.3) |
| BRF | 52/129 (40.3) | 8/126 (6.3) | 91/126 (72.2) | 54/126 (42.9) | 4/126 (3.2) |  | 8/129 (6.2) | 7/129 (5.4) |
| PEL | 96/196 (49) | 37/182 (20.3) | 52/182 (28.6) | 89/182 (48.9) | 4/182 (2.2) |  | 21/196 (10.7) | 35/196 (17.9) |
| TZH | 81/136 (59.6) | 61/100 (61) | 11/100 (11) | 68/100 (68) | 24/100 (24) |  | 7/136 (5.1) | 10/136 (7.4) |

*Prior to determining the prevalence, the hemoglobin distribution was adjusted for altitude, and the distributions of plasma retinol, TfR and ferritin and zinc were adjusted for inflammation following BRINDA.

**Supplemental Figure 2:** Simple correlation matrix of selected variables characterizing nutrient intake density from complementary foods, measures of gut inflammation and permeability, systemic inflammation and concentrations of biochemical indicators of micronutrient status. Abbreviations used: MPO, myeloperoxidase; NEO, neopterin; AAT, α-1 antitrypsin; LMZ, lactulose:mannitol ratio z-score; %Lac-Z, lactulose recovery z score; %Man-Z, mannitol recovery z score; AGP, α-1 acid glycoprotein. Colors are proportional to the correlation coefficient (blue, negative; red positive).

**
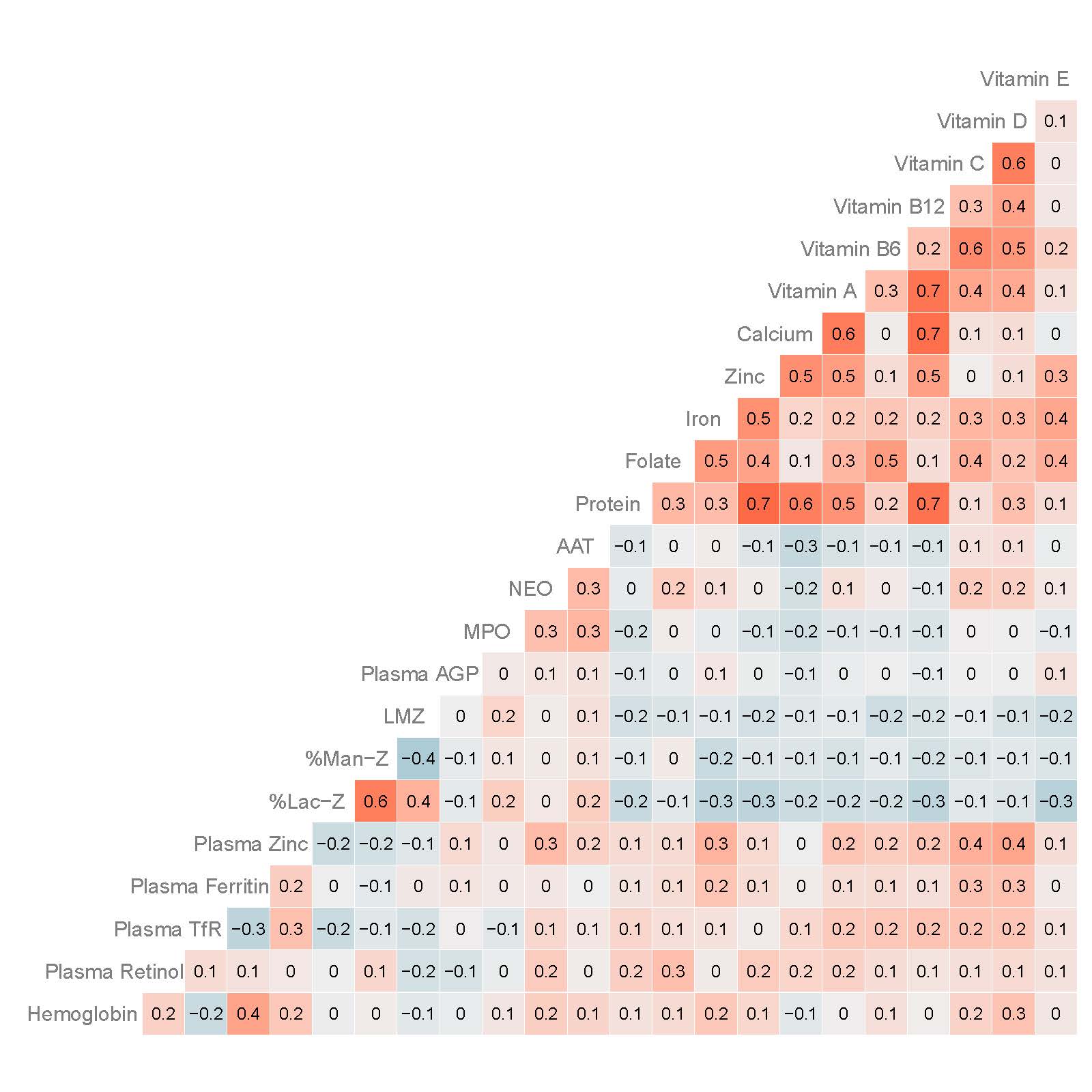
**

**Supplemental Table 2:** Numeric results for the associations depicted in Figure 3 in the main text. Presented are the median effects (95% credibility interval) of each predictor on each response variable adjusted for other associations within the model. Effects are expressed as odds for binary responses; otherwise they are expressed as linear effects

| **Response** | **Predictor** | **Posterior quantile** |
| --- | --- | --- |
|  |  | median (95% credibility interval) |
|  |  | odds |
| Anemia | LMZ | 1.15 (1.01, 1.31) |
|  | Vitamin A density | 1.52 (1.13, 2.02) |
|  | MPO | 1.16 (1.01, 1.34) |
|  | Low retinol | 1.58 (1.16, 2.15) |
|  | High TfR | 2.26 (1.68, 3.09) |
|  | Low ferritin | 2.79 (2.09, 3.68) |
|  | AGP | 1.29 (1.12, 1.49) |
| Low Retinol | LMZ | 1.24 (1.08, 1.45) |
|  | Iron density | 0.74 (0.55, 0.99) |
|  | NEO | 0.75 (0.62, 0.89) |
|  | AGP | 1.58 (1.38, 1.83) |
| Low Ferritin | Iron density | 0.73 (0.57, 0.92) |
|  | NEO | 1.22 (1.04, 1.44) |
|  | AAT | 1.19 (1.03, 1.37) |
|  | AGP | 0.62 (0.54, 0.7) |
| High TfR | Iron density | 0.79 (0.63, 0.99) |
|  | Calcium density | 1.28 (1.01, 1.61) |
|  | MPO | 0.86 (0.74, 0.98) |
|  | AGP | 1.18 (1.03, 1.33) |
| Low Zinc | AAT | 0.83 (0.7, 0.99) |
|  |  | linear effect |
| AGP | NEO | -0.1 (-0.16, -0.03) |
| AAT | Calcium density | -0.15 (-0.24, -0.06) |
|  | Vitamin A density | -0.12 (-0.22, -0.01) |
|  | Vitamin B6 density | -0.08 (-0.16, 0) |
| ZLM | Zinc density | -0.16 (-0.31, -0.02) |
|  | MPO | 0.09 (0.03, 0.15) |
|  | AAT | 0.08 (0.02, 0.14) |
| MPO | Calcium density | -0.22 (-0.33, -0.12) |
|  | Vitamin A density | -0.16 (-0.27, -0.04) |
|  | Protein density | -0.13 (-0.22, -0.04) |
|  | Vitamin B6 density | -0.09 (-0.18, 0) |
|  | Vitamin B12 density | 0.27 (0.14, 0.39) |
| NEO | Vitamin A density | -0.16 (-0.25, -0.06) |

Abbreviations used: AAT, alpha-1-antitrypsin; AGP, alpha-1-acid glycoprotein; LMZ, lactulose:mannitol ratio z-score; MPO, myeloperoxidase; NEO, neopterin; TfR, transferrin receptor

**Supplemental Figure 3**: All associations tested in the multivariate DAG. The width of the arc is proportional to the mean effect. Positive associations are shown in red and negative associations in blue. Arcs for which the 95% credibility interval did not include zero are solid and those that did include zero are indicated with dotted lines. Folate and vitamin C (grey ellipse) were dropped from the model because no univariate associations were significant, and folate is correlated with Vitamins B6 and B12. Abbreviations used: MPO, myeloperoxidase; NEO, neopterin; AAT, α-1 antitrypsin; LMZ, urinary lactulose:mannitol ratio z-score; AGP, α-1 acid glycoprotein.


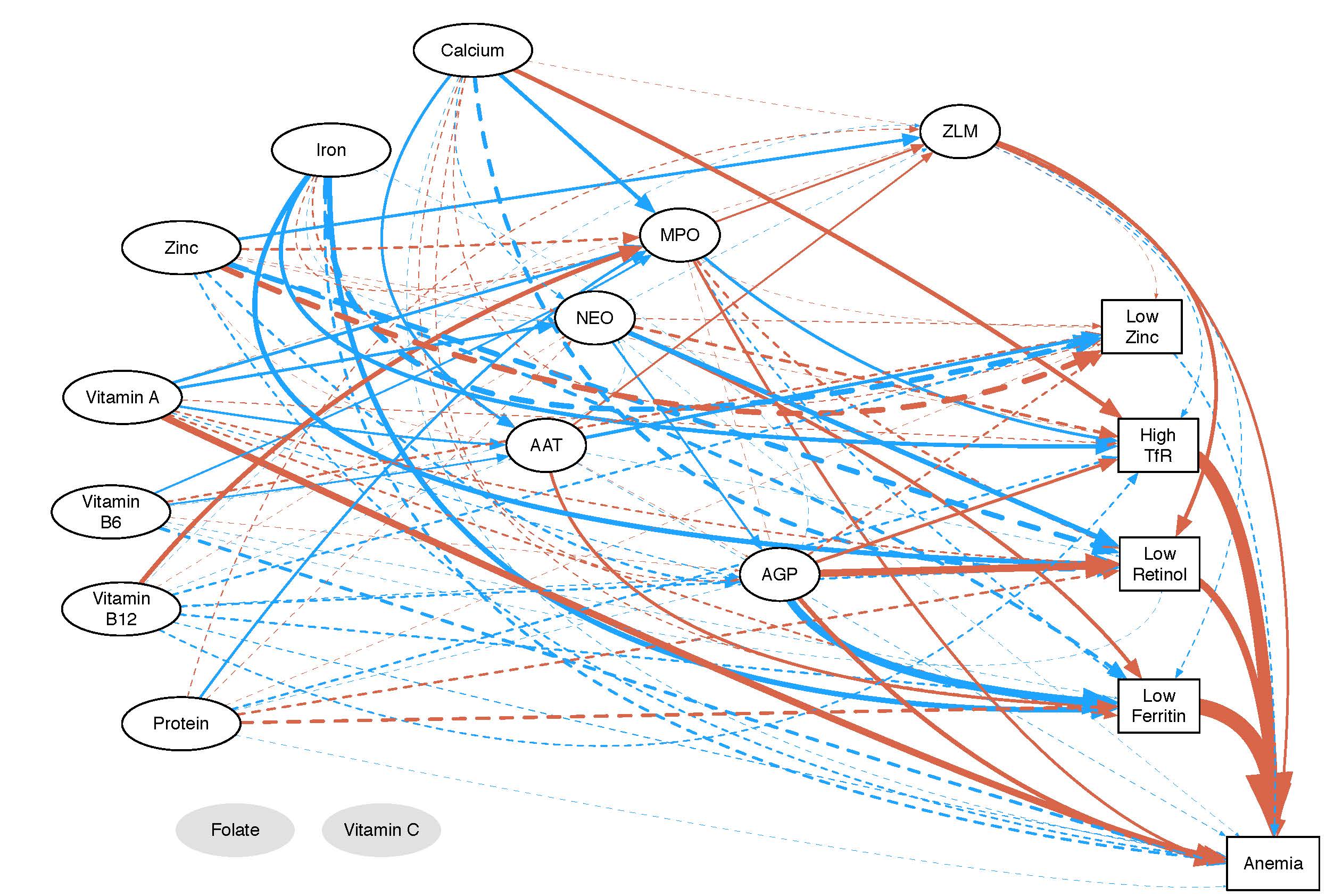


**Supplemental Figure 4**: Comparison of generalized mixed models of micronutrient status as a function of nutrient density, EED biomarkers and including fever as an additional indicator of systemic inflammation. The coefficients of four different models are shown, if fever is not included (the model in the main text, grey open circles), a binary variable of whether or not the child experienced fever in the 7 days preceding the blood draw (yellow closed circles), a binary variable of whether or not the child experienced fever in the 7 days after the blood draw (green triangles) or the proportion of days (x10) when the mother reported that the child had a fever in the 9 to 15 month period (blue diamonds). There are no differences in coefficients between models whether fever is included or not.
